# The Eyelid Meibomian Gland Deficiency in Fucosyltransferase 1 Knockout Mice

**DOI:** 10.3390/ijms23169464

**Published:** 2022-08-21

**Authors:** Chang Ho Yoon, Jin Suk Ryu, Jung Hwa Ko, Yeon Kyung Kim, Jang-Hee Oh, Jin Ho Chung, Joo Youn Oh

**Affiliations:** 1 Department of Ophthalmology, Seoul National University College of Medicine, 103 Daehak-ro, Jongno-gu, Seoul 03080, Korea; 2 Laboratory of Ocular Regenerative Medicine and Immunology, Biomedical Research Institute, Seoul National University Hospital, 101 Daehak-ro, Jongno-gu, Seoul 03080, Korea; 3 Department of Dermatology, Seoul National University College of Medicine, 103 Daehak-ro, Jongno-gu, Seoul 03080, Korea

**Keywords:** eyelid, fucosylation, fucosyltransferase 1, glycosylation, inflammation, meibomian gland, ocular surface, oxidative stress

## Abstract

To investigate the effect of fucosyltransferase (FUT) 1-mediated fucosylation on meibomian glands (MG), we first confirmed that FUT1 and its fucosylated products were expressed in the eyelid, conjunctiva and skin in wild-type (WT) mice, whereas their mRNA and protein levels were downregulated in Fut1 knock-out (KO) mice. We then evaluated age-dependent changes in the total and acinar areas of MG, meibocyte differentiation, lipid synthesis, and eyelid inflammation and oxidative stress in Fut1 KO and WT mice. Results show that both the total and acinar areas of MG were smaller in Fut1 KO mice than in WT mice in all evaluated age groups. Meibocyte differentiation, lipid-producing capacities and the enzyme levels responsible for lipid synthesis were reduced in Fut1 KO mice, compared to WT controls. The levels of pro-inflammatory cytokines and oxidative-stress-related markers were elevated in the eyelids and MG of FUT1 KO mice. These findings demonstrate the physiologic function of FUT1-mediated fucosylation in MG development and function, and indicate its potential role in ocular surface homeostasis.

## 1. Introduction

Fucosylation, a type of glycosylation, is an enzymatic process of transferring a fucose residue to acceptor molecules such as oligosaccharides, proteins and lipids, and is performed by fucosyltransferases (FUTs) [1]. Fucosylated carbohydrate moieties, the products of fucosylation, are increasingly recognized as vital to a wide range of physiological and pathological processes, including fertilization, cell differentiation, tissue development, immune system regulation and malignancy [2,3,4,5]. Given the abundance of glycosylated mucins, glycosaminoglycans and glycosphingolipids in tears, as well as in conjunctival and corneal epithelia, it has long been considered that glycosylation might play a role in the regulation of ocular surface homeostasis [6,7,8,9]. However, most of these findings are observational and thus are in need of interventional studies to determine the functional consequences of glycosylation in the ocular surface.

Our group previously observed that FUT1 deficiency induced corneal epithelial defects and opacity at least in part by activating CD4^+^ T cells, but not by directly affecting lacrimal glands [10]. Rather, the aqueous tear production from lacrimal glands was increased in Fut1 knock-out (KO) mice, indicating reflex tearing in response to the ocular surface disruption. The tear film is critical for maintaining ocular surface homeostasis and is composed of a lipid layer secreted by meibomian glands (MGs), as well as an aqueous layer produced by lacrimal glands. The tear film lipid deficiency associated with MG dysfunction (MGD) is one of the causes of evaporative dry eye disease (DED), while the reduced tear secretion due to lacrimal gland damage can cause aqueous-deficient DED [11].

MGs are holocrine sebaceous glands located in both the upper and lower eyelids, and consist of meibocytes secreting meibum, a lipid component of tears that protects the ocular surface from aqueous tear evaporation, environmental injuries and infections [12]. As such, MGs play an important role in the maintenance of ocular surface homeostasis. Moreover, in recent years, MGD has been identified as the most common cause of DED, representing approximately up to 70% of DED cases worldwide [11,13].

Despite the importance of MGs in ocular surface physiology and pathology, the role of fucosylation and its fucosylated products in MG development and function remains poorly elucidated. In this study, we investigated the effects of FUT1-mediated fucosylation on MGs with age using Fut1 KO mice.

## 2. Results

### 2.1. Expression of FUT1 and Its Fucosylated Products in the Eyelid and MGs

To determine the biological relevance of fucosylation in the eyelid MG, we evaluated whether FUT1 and its fucosylated products were expressed in the eye.

**Figure 1:**
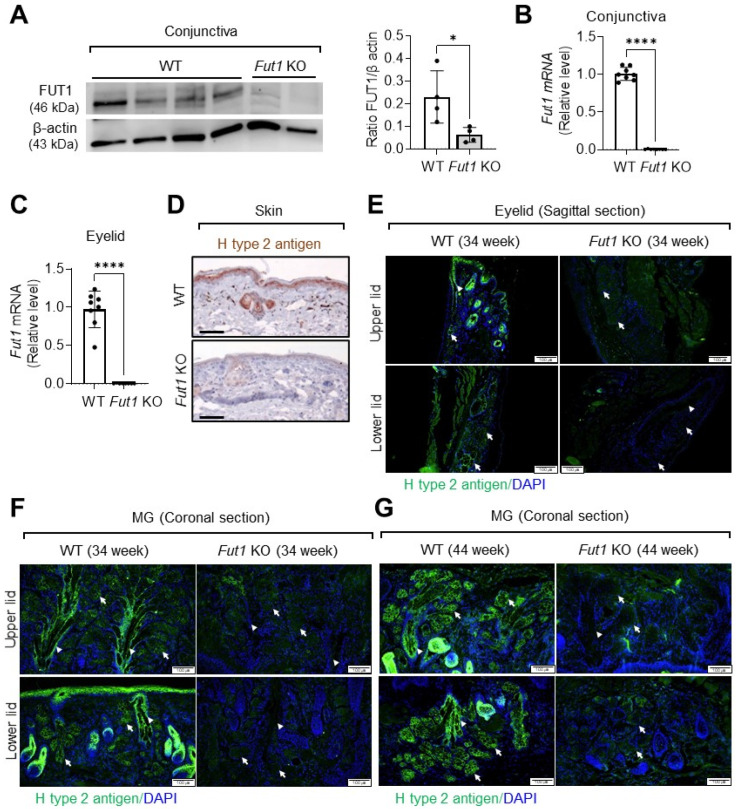
Loss of FUT1 and fucosylated products in the skin, conjunctiva, eyelid and MG in Fut1 KO mice vs. WT mice. (**A**) Representative Western blot image and quantitative densitometric analysis of FUT1 and β actin in the conjunctiva in WT and Fut11 KO mice. (**B**,**C**) Real-time RT-PCR for the Fut1 gene in the conjunctiva (**B**) and eyelid (**C**). The mRNA levels in Fut1 KO mice are presented as fold changes relative to those in WT mice. (**D**) Immunostaining of H type 2 antigen in the skin in WT and Fut1 KO mice. Scale bar: 50 μm. (**E**–**G**) Immunofluorescence staining of H type 2 antigen in the eyelid (**E**, sagittal section) and in MG (**F**,**G**, coronal section) in 34- and 44-week-old WT and Fut1 KO mice. The white arrows and arrowheads indicate the acinar and ductal epithelial cells of MG, respectively. Mean values ± SD are presented, where each dot represents the data from an individual mouse. * *p* < 0.05, **** *p* < 0.0001, as analyzed by unpaired *t*-test.

First, we confirmed that the protein and RNA levels of FUT1 were significantly lost in the conjunctiva and eyelid in Fut1 KO mice relative to wild-type (WT) controls, as assessed by a Western blot analysis (Figure 1A) and real-time RT-PCR (Figure 1B,C), respectively.

Then, we found that the blood group H type 2 antigen, which is an important fucose-containing glycan structure synthesized by FUT1 [14], was expressed in the skin (Figure 1D), eyelid (Figure 1E) and MGs (Figure 1F,G) in WT mice as demonstrated by immunohistochemistry, while its expression was markedly decreased in Fut1 KO mice. Particularly, the expression of H type 2 antigen was prominent in the acinar and ductal epithelial cells within MGs in WT mice, while its expression was almost completely abolished in Fut1 KO mice (Figure 1F,G).

### 2.2. Effects of FUT1 Deficiency on MG Development and Function with Age

To investigate the effects of FUT1 on the development and function of MG, we next assessed the changes in the total and acinar areas of MGs and the levels of meibocyte differentiation and lipid synthesis in Fut1 KO mice with age, and compared them with those in WT mice.

**Figure 2:**
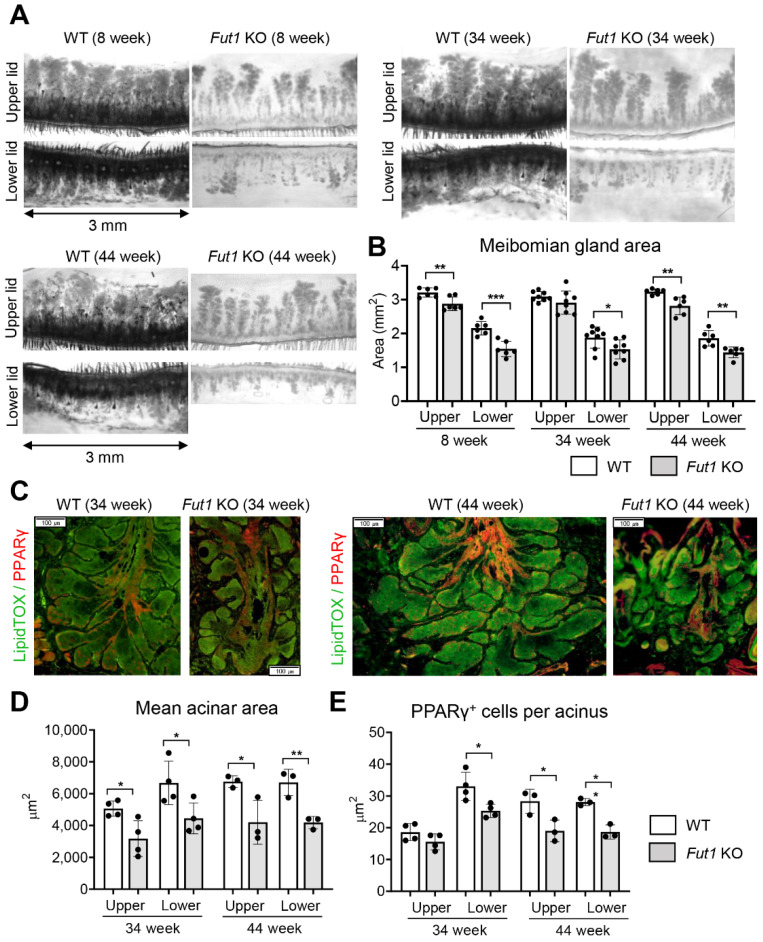
MG deficiency in Fut1 KO mice. (**A**) Representative transillumination meibography images of the upper and lower eyelids in WT and Fut1 KO mice at 8, 34 and 44 weeks of age. (**B**) Quantitation of the total MG area in the upper and lower eyelids. (**C**) Representative LipidTOX and PPARγ immunostaining of coronal sections of MGs in WT and Fut1 KO mice at 34 and 44 weeks of age. Scale bar: 100 μm. (**D**,**E**) Quantitation of the mean LipidTOX^+^ MG acinar area and the number of PPARγ^+^ meibocytes per acinus in the upper and lower eyelids in 34- and 44-week-old WT and Fut1 KO mice. Mean values ± SD are presented, where each dot represents the data from an individual mouse. * *p* < 0.05, ** *p* < 0.01, *** *p* < 0.001, as analyzed by unpaired *t*-test or Mann–Whitney U test.

Transillumination meibography revealed that the total area of MGs was approximately two times larger in the upper eyelids than in the lower eyelids in 8-week, 34-week and 44-week-old Fut1 KO mice, and that the MG areas in both upper and lower eyelids did not change with age (Figure 2A,B). Remarkably, the total MG area in upper and lower eyelids was significantly smaller in Fut1 KO mice, compared to WT controls, in all age groups (Figure 2A,B).

Similar observations were made with the acinar area of MGs and the number of meibocytes. LipidTOX and PPARγ were used as markers for MG acini and meibocytes, respectively [15]. Immunofluorescence staining showed that both the mean acinar area (marked by LipidTOX) and the meibocyte number (marked by PPARγ) were significantly decreased in the upper and lower eyelids in Fut1 KO mice, compared to their counterparts in WT mice at both 34 and 44 weeks of age (Figure 2C–E). 

Collectively, the data demonstrate that the total and acinar areas of MG, meibocyte differentiation and lipid production capacity are reduced in Fut1 KO mice, but the reduction does not worsen with age.

### 2.3. Effects of FUT1 Deficiency on Genes Related to Inflammation and Meibocyte Development

**Figure 3:**
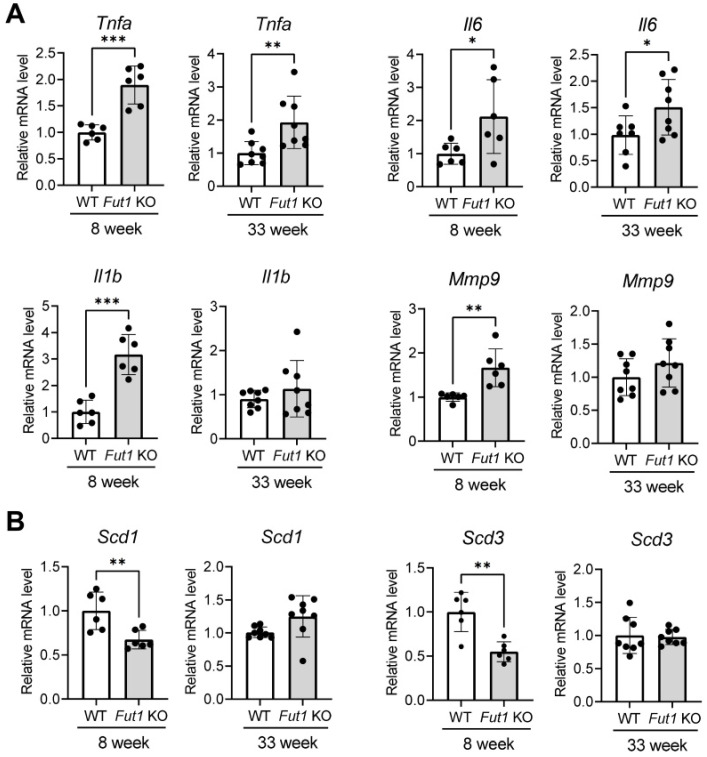
Expression of inflammation- and meibocyte-differentiation-related markers in the eyelid of FUT1 KO mice vs. WT mice. (**A**) Real-time RT-PCR for pro-inflammatory cytokines (TNF-α, IL-1β and IL-6) and enzyme (MMP9) in the eyelid of mice at 8 and 33 weeks of age. (**B**) Real-time RT-PCR for Scd1 (a marker for pre-sebocytes) and Scd3 (a marker for mature sebocytes) in the eyelid of mice at 8 and 33 weeks of age. The mRNA levels in Fut1 KO mice are presented as fold changes relative to those in WT mice (mean values ± SD). Each dot represents the data from an individual mouse. * *p* < 0.05, ** *p* < 0.01, *** *p* < 0.001, as analyzed by unpaired *t*-test or Mann–Whitney U test.

Fucosylation plays an important role in the regulation of inflammation and the immune response [10,14]. The eyelid inflammation often accompanies MGD in patients and has been reported to impair the structure and function of MGs [16,17]. Hence, we next evaluated whether the eyelid inflammation might be increased in Fut1 KO mice. The levels of TNF-α and IL-6 in the eyelids were significantly higher in both 8-week and 33-week-old Fut1 KO mice than in WT mice of the same age, as measured by real-time RT-PCR (Figure 3A). Similarly, the mRNA levels of IL-1β and matrix metallopeptidase (MMP)9 were increased in 8-week-old Fut1 KO mice, compared to 8-week-old WT mice (Figure 3A).

We additionally analyzed the eyelids for stearoyl-CoA desaturase (SCD), a microsomal enzyme involved in lipid synthesis critical for normal MG function [18,19,20]. The mRNA levels of both Scd1 (a marker for pre-sebocytes) and Scd3 (a marker for mature sebocytes) were significantly downregulated in 8-week-old Fut1 KO mice, relative to 8-week-old WT mice, while they were not different between 33-week-old Fut1 KO and WT mice (Figure 3B).

### 2.4. Effects of FUT1 Deficiency on Genes Related to Oxidative-Stress-Related Markers

**Figure 4:**
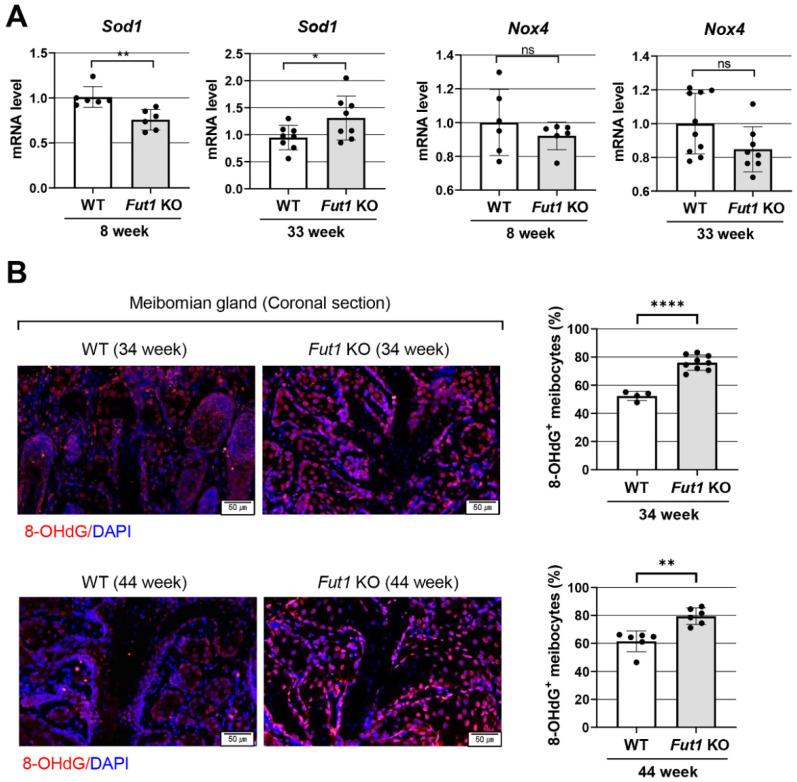
Expression of oxidative-stress-related markers in the eyelid of Fut1 KO mice vs. WT mice. (**A**) Real-time RT-PCR analysis for an antioxidant gene Sod1 and an oxidative-stress-related gene Nox4 in the eyelid of Fut1 KO and WT mice at 8 and 33 weeks of age. Shown are the mRNA levels in Fut1 KO mice relative to those in WT mice (mean values ± SD). A dot represents the data from a single individual animal. (**B**) Representative 8-OHdG immunostaining image of MG coronal sections and quantitative analysis of 8-OHdG^+^ meibocytes in WT vs. Fut1 KO mice at 34 and 44 weeks of age. Scale bar: 50 μm. Mean values ± SD are presented, where each dot represents the data from the upper or lower eyelid of a single individual animal. * *p* < 0.05, ** *p* < 0.01, **** *p* < 0.0001, ns: not significant, as analyzed by unpaired *t*-test or Mann–Whitney U test.

We furthermore tested whether oxidative stress might be involved in the structural disruption and dysfunction of MGs in Fut1 KO mice. The mRNA level of a gene encoding an antioxidant enzyme superoxide dismutase (SOD) 1 was lower in Fut1 KO mice at 8 weeks of age, while it was higher in the eyelids of Fut1 KO mice at 33 weeks, compared to WT mice (Figure 4A). Immunofluorescence staining of MGs for an oxidative stress marker, 8-hydroxydeoxyguanosine (8-OHdG), revealed that the number of 8-OHdG^+^ meibocytes was significantly higher in Fut1 KO mice, compared to WT mice, at both 34 and 44 weeks (Figure 4B). There was, however, no difference in the level of Nox4, a gene encoding NADPH oxidase 4 which produces reactive oxygen species (ROS), between Fut1 KO and WT mice either at 8 or 33 weeks of age (Figure 4A).

## 3. Discussion

Our data demonstrate that FUT1 and its fucosylated products are expressed in the eyelid and MG, and FUT1 deficiency impedes the eyelid MG development and function in mice. Since the disruption of MGs in Fut1 KO mice did not progress until 44 weeks of age, it is likely that FUT1 deficiency affects the development of MG but does not contribute to age-dependent MG dropout and meibum loss, common findings in age-related DED in old patients [21,22,23]. Given, however, that mice between 34 and 44 weeks of age are considered middle-aged, not old-aged, from an aging perspective, it would be necessary to confirm these results in older mice.

In the previous study, we observed higher numbers of corneal epithelial defects in Fut1 KO mice, both in a steady state and under desiccating stress, indicative of more severe DED, despite the fact that aqueous tear production from lacrimal glands or mucin-secreting goblet cells in the conjunctiva did not decrease defects in Fut1 KO mice [10]. This manifestation of severe DED in Fut1 KO mice might be due to the perturbation of MGs and their function associated with FUT1 deficiency, as we observed in the present study. In fact, MGs play a key role in preventing DED by secreting a lipid layer of tear film and thereby blocking the aqueous tear evaporation [24]. MGD was recently considered the most common cause of DED, accounting for 70% of DED cases [11,13]. Yet there is limited knowledge on MG development, homeostasis and pathology. Our findings suggest a potential role of FUT1 and its fucosylated products in MG development and function.

In line with our study, previous studies have reported an association of altered glycosylated products with the development of MGD and DED. Sun et al. [25]. demonstrated that abnormal hyaluronan synthesis induced precocious and exacerbated MG morphogenesis, leading to dysmorphic eyelids and MGs in hyaluronan synthase KO mice. Pan et al. [26]. revealed that heparan sulfate modification is required for lacrimal gland development. Additionally, Mantelli et al. [27] showed the differential expression profiles of glycogenes (genes involved in biosynthesis of glycoconjugates) in the conjunctiva between normal subjects and DED patients. These observations made by others, along with our findings, implicate glycosylation in MG development and DED pathogenesis, providing a novel therapeutic target for MGD and DED.

There are three possible mechanisms responsible for MGD in Fut1 KO mice. First, FUT1-mediated fucosylation might be directly involved in the differentiation and proliferation of meibocytes required for lipid production. In support of this possibility, the number of PPARγ^+^ cells in MGs was significantly decreased in Fut1 KO mice in our study. PPARγ is characteristically expressed in lipid-synthesizing cells and serves as a biomolecular marker for differentiated meibocytes [15]. Moreover, the levels of both Scd1 (expressed in pre-sebocytes) and Scd3 (expressed exclusively in mature sebocytes) [18,19] were also downregulated in the MGs of Fut1 KO mice, indicating the loss of meibocytes. In line with our findings, FUT1 knockdown has been shown to inhibit human epidermoid carcinoma cell proliferation [28], keratinocyte migration [29] and synovial fibroblast proliferation [30].

Second, the inflammation associated with FUT1 deficiency might exert detrimental effects on MG. Inflammation in the eyelids and ocular surface is a major aggravating factor in MGD [31,32,33]. It is well-known that fucosylation plays important indirect roles in immune responses by regulating cell adhesion, migration, differentiation, polarization and cell-to-cell interaction [14]. For example, we previously found that the Th1 immune response was upregulated in the ocular surface and the ocular draining lymph nodes in Fut1 KO mice [10]. It was also reported that fucosylation inhibition induced the upregulation of MHC class II and co-stimulatory molecules on dendritic cells [34]. In agreement with these findings, the expression of pro-inflammatory cytokines and enzymes was elevated in the eyelids of Fut1 KO mice in the present study. Further studies would be necessary to investigate how inflammation and immune responses affect meibocyte differentiation and meibomian gland function.

Third, it is also possible that oxidative stress might contribute to MGD in Fut1 KO mice. In our study, higher numbers of 8-OHdG-expressing meibocytes were found in Fut1 KO mice than in WT mice. In addition, the expression of SOD1, an antioxidant enzyme, was significantly reduced in the eyelids of 8-week-old Fut1 KO mice compared to WT counterparts. Since SOD1 attenuates ocular inflammation by scavenging ROS [35], a lower level of SOD1 in Fut1 KO mice might lead to eyelid inflammation and MGD. Interestingly, SOD1 expression was rather increased in 33-week-old Fut1 KO mice, which may be reflective of the reactive process subsequent to upregulated inflammation. Further mechanistic studies would be needed to explain this discrepancy.

In conclusion, our study indicates that FUT1 deficiency impairs meibocyte differentiation/proliferation, reduces the total and acinar areas of MG, and induces eyelid inflammation and oxidative stress, leading to MGD. Homeostatic control of fucosylation and fucosylated products in the ocular surface and eyelids may be a possible therapeutic strategy for MGD and DED.

## 4. Materials and Methods

### 4.1. Animals

The animal experiment protocol was approved by the Institutional Animal Care and Use Committee of Seoul National University Biomedical Research Institute (no. 19-0240-S1A1). Animal experiments were performed in accordance with the Association for Research in Vision and Ophthalmology (ARVO) Statement for Use of Animals in Ophthalmic Vision and Research. Fut1 KO mice (C57BL/6 background, B6.129-Fut1tm1Sdo/J) and C57BL/6 mice were purchased from Jackson Laboratory (Bar Harbor, ME, USA) [36]. Genetic confirmation of Fut1 gene knockdown was performed following the genotyping protocols from Jackson Laboratory [36]. The mice were housed in a specific pathogen-free environment and freely fed a normal diet of laboratory rodent chow and water. Altogether, 8-week, 33-week, 34-week and 44-week-old Fut1 KO mice (B6.129-Fut1tm1Sdo/J) were used in this study, and C57BL/6 mice of the same age were used as wild-type (WT) controls.

### 4.2. Transillumination Meibography

MGs were visualized, and their total area was evaluated by transillumination meibography, as previously described [37]. Briefly, after excision of the upper and lower eyelids, their skin was gently removed. Each tissue was positioned between two microscopic slides and subsequently placed over a wide-spectrum white light source (LED plate). Images of the light transmitted through the tissue were taken using a microscope equipped with an infrared filter (Hoya R72, TOKINA Co., Ltd., Tokyo, Japan) and a Charge-Coupled Device camera (acA1600-20 µm, Basler Inc., Ahrensburg, Germany). Photographs of the central 3 mm area were analyzed using ImageJ software (v. 1.8.0; NIH Image, Bethesda, MD, USA), wherein MGs were selected semi-automatically using the wand tool (tolerance range of 5 to 100), and their areas were measured.

### 4.3. Immunohistochemistry

The expression of FUT1-fucosylated products (blood types H2 antigen), neutral lipid (LipidTOX™) and peroxisome-proliferator-activated receptor γ (PPARγ) was evaluated in the eyelids or skin using immunohistochemistry. The tissues were fixed in 10% formaldehyde, sliced into 4 μm-thick sections and subjected to immunostaining. The primary antibodies used were anti-blood type H2 antigens (BRIC231, #sc-59467 Santa Cruz Biotechnology, Dallas, TX), neutral lipids (HCS LipidTOX™ Deep Green Neutral Lipid Stain, #H34475; Invitrogen, Carlsbad, CA, USA), PPARγ (a marker for meibocytes, #ab59256, Abcam, Cambridge, MA, USA) and 8-OHdG (#GTX41980, GeneTex, Irvine, CA, USA).

The LipidTOX^+^ MG acinar area and the number of PPARγ^+^ cells per acinus were measured in the ImageJ software. The average values of the acinar area and the PPARγ^+^ cell number in the 5 largest acini connected to the central duct were used for comparison between groups.

### 4.4. Western Blot

The expression of FUT1 was analyzed in the conjunctiva using a Western blot. For protein extraction, the conjunctival tissue was chopped with scissors and sonicated on ice in RIPA buffer (Biosesang, Seongnam, Korea) supplemented with Halt^TM^ Protease Inhibitor Cocktail (#78438, Thermo Fisher Scientific, Waltham, MA, USA). After centrifugation at 12,000 rpm at 4 °C for 20 min, the protein concentration in the tissue lysates was determined using the Bradford assay. After heating at 95 °C for 5 min, 50 μg of total protein was loaded into 8–16% SDS-PAGE gel (Komabiotech, Seoul, Korea) for 2 h at 150 V, transferred to a PVDF membrane (Thermo Fisher Scientific) and stained with antibodies against FUT1 (1:500, #sc-21963, Santa Cruz Biotechnology) and β actin (1:1000, #sc-47778, Santa Cruz Biotechnology) at 4 °C overnight.

### 4.5. Real-Time Reverse Transcription Polymerase Chain Reaction (RT-PCR)

The mRNA levels of inflammation-, lipid-synthesis- and oxidative-stress-related genes, along with the Fut1 gene, were evaluated using real-time RT-PCR. For RNA extraction, the tissue was cut into small pieces with scissors, lysed in RNA isolation reagent (RNA-Bee, Tel-Test Inc., Friendswood, TX, USA) and homogenized using a sonicator (Ultrasonic Processor, Cole Parmer Instruments, Vernon Hills, IL, USA). Total RNA was extracted from tissue lysates with an RNeasy Mini kit (Qiagen, Valencia, CA, USA) and converted to first-strand cDNA via reverse transcription using a High Capacity RNA-to-cDNA kit (Applied Biosystems, Carlsbad, CA, USA). The cDNA was subjected to real-time PCR amplification for FUT1, TNF-α, IL-1β, IL-6, MMP9, SCD1, SCD3, SOD1 and NOX4 on an ABI 7500 Real Time PCR System (Applied Biosystems). All of the PCR probe sets were purchased from Applied Biosystems (TaqMan Gene Expression Assay kits). Mouse-specific GAPDH was used as an internal control. The data were normalized to GAPDH and expressed as fold changes relative to controls.

### 4.6. Statistical Analysis

Prism software v.9.3.1 (GraphPad, La Jolla, CA, USA) was used for statistical tests. After the normal distribution of data was determined by the D′Agostino and Pearson or the Shapiro–Wilk test, the data were analyzed by an unpaired *t*-test or a Mann–Whitney U test. All data were presented as mean ± SD, and *p* values < 0.05 were considered statistically significant.

## Data Availability

The data presented in this study are available within the article.
